# Structural characterization of melatonin as an inhibitor of the Wnt deacylase Notum

**DOI:** 10.1111/jpi.12630

**Published:** 2020-01-24

**Authors:** Yuguang Zhao, Jingshan Ren, James Hillier, Margaret Jones, Weixian Lu, Edith Yvonne Jones

**Affiliations:** ^1^ Division of Structural Biology Wellcome Centre for Human Genetics University of Oxford Oxford UK

**Keywords:** crystal structure, fragment screen, melatonin, Notum, Wnt signalling

## Abstract

The hormone melatonin, secreted from the pineal gland, mediates multiple physiological effects including modulation of Wnt/β‐catenin signalling. The Wnt palmitoleate lipid modification is essential for its signalling activity, while the carboxylesterase Notum can remove the lipid from Wnt and inactivate it. Notum enzyme inhibition can therefore upregulate Wnt signalling. While searching for Notum inhibitors by crystallographic fragment screening, a hit compound N‐[2‐(5‐fluoro‐1H‐indol‐3‐yl)ethyl]acetamide that is structurally similar to melatonin came to our attention. We then soaked melatonin and its precursor N‐acetylserotonin into Notum crystals and obtained high‐resolution structures (≤1.5 Å) of their complexes. In each of the structures, two compound molecules bind with Notum: one at the enzyme's catalytic pocket, overlapping the space occupied by the acyl tail of the Wnt palmitoleate lipid, and the other at the edge of the pocket opposite the substrate entrance. Although the inhibitory activity of melatonin shown by in vitro enzyme assays is low (IC_50_ 75 µmol/L), the structural information reported here provides a basis for the design of potent and brain accessible drugs for neurodegenerative diseases such as Alzheimer's disease, in which upregulation of Wnt signalling may be beneficial.

## 1 INTRODUCTION

Melatonin, N‐acetyl‐5‐methoxytryptamine, is an ancient and evolutionarily conserved molecule used by most living organisms to maintain their circadian rhythms.^1^ Human melatonin is centrally produced by the pineal gland at night and released into the blood stream as well as the cerebrospinal fluid of the central nervous system. Apart from its well‐known circadian rhythm roles,^2^ melatonin may have many other biological functions, such as regulating energy metabolism,^3^ blood pressure,^4^ reproduction,^5^ immune response,^6^ apoptosis^7^ and autophagy,^8^ and exhibits anti‐inflammatory,^9^ anti‐cancer^10^ and anti‐ageing^11^ activities. Melatonin also shows antioxidant effects in vitro, although the in vivo relevance remains controversial.^12^

Being a privileged amphiphilic molecule, melatonin can rapidly access neurons and other tissues^13^ to protect neurons,^14^, ^15^ maintain bone health and alleviate osteoporosis.^16^, ^17^ Multiple signalling pathways may be targeted by melatonin, including Wnt signalling.^18^ Interestingly, Wnt signalling also has neuroprotective,^19^, ^20^, ^21^ bone mass maintenance and regeneration roles.^22^ It has been shown that melatonin can activate Wnt/β‐catenin signalling in neuron cells^23^, ^24^ and osteoblasts.^25^

The ancient and evolutionarily conserved Wnt signalling pathway plays multiple roles in animal embryonic development as well as adult tissue homeostasis and regeneration,^26^ and loss of function may contribute to neurodegenerative diseases such as Alzheimer's disease.^27^ Wnt ligands are post‐translationally modified by a palmitoleate moiety attached to a serine,^28^ while the recently discovered Wnt deacylase Notum can remove this moiety and acts as a secreted feedback antagonist.^29^ Notum belongs to the α/β‐hydrolase superfamily of enzymes, and its structure reveals a large hydrophobic pocket at the active site,^29^ suggesting its enzyme activity can be blocked by small molecule inhibitors, and inhibition of Notum can increase bone density^30^ and may have the potential to rejuvenate stem cells.^31^

Activity‐based small molecule screens for Notum inhibitors have been performed.^32^ We recently reported the development of 2‐phenoxyacetamides as Notum inhibitors.^33^ One of the biggest limitations of these inhibitors is their inability or limited ability to cross the blood‐brain barrier, even though they are found to be effective in vitro.^30^, ^33^ In order to identify new chemically divergent small molecules and generate structural information for rational design of new inhibitors, we used a crystallography‐based fragment screening approach.^34^ One of the hit molecules, N‐[2‐(5‐fluoro‐1H‐indol‐3‐yl)ethyl]acetamide, is chemically very similar to melatonin. We then tested melatonin and its precursor N‐acetylserotonin and obtained high‐resolution crystal structures of their complexes with Notum.

## 2 MATERIALS AND METHODS

### 2.1 Reagents

N‐[2‐(5‐fluoro‐1H‐indol‐3‐yl)ethyl]acetamide was purchased from Molport, melatonin from Fluorochem, N‐acetylserotonin from Cambridge Bioscience and the lipase enzyme‐substrate OPTS (8‐octanoyloxypyrene‐1,3,6‐trisulfonate) from Sigma.

### 2.2 Protein production

Human Notum protein was produced in mammalian cells. HEK293S GNTI‐ cells^35^ were cultured in DMEM (high glucose, Sigma) with 1 mmol/L glutamine, 1× nonessential amino acids and 10% FBS (Invitrogen) at 37°C with 5% CO_2_. For large scale production, cells were grown in expanded surface roller bottles (Greiner).

We cloned the human Notum enzyme core sequence comprising S81‐T451 with a C330S mutation (Notum_core_)^29^ into a stable cell line vector pNeo_sec.^36^ HEK293S GNTI‐ cells^35^ were co‐transfected with pNeo‐Notum_core_ and a PhiC31 integrase expression vector (pgk‐phiC31).^37^ The polyclonal cell population resulting from G418 (1 mg/mL) selection was cultured to produce protein. For protein purification, the dialysed conditioned media were incubated with talon beads for 1 hour at 16°C. The beads were collected and washed with 10 mmol/L imidazole PBS and eluted with 200 mmol/L imidazole PBS. To remove flexible glycans, which can prevent crystal growth, the protein was deglycosylated with endo‐β‐N‐acetylglucosaminidase F1 (37°C, 1 hour) and further purified by size‐exclusion chromatography (Superdex 200 16/60 column, GE Healthcare) in 10 mmol/L Hepes, pH 7.4, 150 mmol/L NaCl buffer.

### 2.3 Crystallization, fragment‐based screening and structure determination

Notum protein was concentrated to 5 mg/mL and crystallized using the sitting drop vapour diffusion method at 21°C^38^ in 96‐well Swissci/MRC plates. The crystallization drops contained 200 nL of Notum protein and 100 nL of reservoir solution of 1.5 M ammonium sulphate and 0.1 M sodium citrate, pH 4.2.

Fragment‐based screening was carried out using the XChem platform and beamline I04‐1 at the Diamond Light Source (http://www.diamond.ac.uk/Instruments/Mx/Fragment-Screening). In preparation for the screen, the crystallization drops were imaged using a Rock Imager 1000 (Formulatrix, Inc) and analysed with TeXRank^39^ to score crystal quality. Manual annotation, based on further visual inspection, was used to identify the regions of selected drops into which fragment solutions could best be dispensed to minimize direct disruption of crystals.

We used the Diamond‐SGC poised library (DSPL),^40^ for which all the "Poised" fragments contain at least one functional group which can be synthesized using a well‐characterized reaction. The library compounds were contained within a 1536 well ECHO plate at 500 mmol/L concentration (in DMSO). Using an ECHO liquid handler (Labcyte INC) with acoustic droplet ejection technology, 60 nL of the individual fragment compounds was dispensed and recorded as described.^41^ After one hour of soaking, the crystals were harvested using a Shifter x‐y device (Oxford Lab Technologies) under the control of SoakDB (an Excel interface to a sqlite database file), and data were exported to XChemExplorer (a data management and workflow tool for the parallel determination of protein‐ligand structures, developed by the I04‐1 XChem team and available at http://tkrojer.github.io/XChemExplorer/). The X‐ray diffraction data were collected on beamline I04‐1, in automated unattended mode. Twenty apo datasets (soaked with 20% DMSO) were used for background electron density extraction by PanDDA (Pan‐Dataset Density Analysis),^42^ and the datasets with PanDDA positive hits were further confirmed and refined with REFMAC.^43^ The crystal structures were validated using MolProbity.^44^ Figures were prepared with the PyMOL Molecular Graphics System (Schrödinger, LLC.).

For manual crystal soaking with nonlibrary compounds, crystals were grown in the same way as described above, and compounds were mixed with crystal growing reservoir solution at a final concentration of 10 mg/mL and soaked for 1 hour. Data were collected at Diamond beamline I04 or I04‐1.

Data collection and refinement statistics are shown in Table 1.

**Table 1:** Data collection and refinement statistics

	Notum_ Fragment 106	Notum_Melatonin	Notum_N‐acetylserotonin
PDB ID code	6TR7	6TR5	6TR6
Ligand code	HWH	ML1	ASE
Data collection			
X‐ray source (Diamond)	I04‐1	I04‐1	I04
Wavelength (Å)	0.92819	0.92819	1.0400
Space group	P2_1_2_1_2_1_	P2_1_2_1_2_1_	P2_1_2_1_2_1_
Cell dimensions			
*a*, *b*, *c* (Å)	60.3, 73.3, 79.0	60.3, 73.2, 79.0	59.5,72.7, 77.9
α, β, γ (°)	90, 90, 90	90, 90, 90	90, 90, 90
Resolution (Å)	1.47 (1.50‐1.47)^a^	1.51 (1.54 −1.51)	1.35 (1.37 ‐ 1.35)
*R*_merge_	0.062 (‐‐‐)	0.065 (‐‐‐)	0.045 (‐‐‐)
*I*/σ*I*	12.7 (1.1)	15.0 (1.3)	23.6 (1.0)
Completeness (%)	100 (98.6)	99.6 (94.1)	97.3 (73.9)
Redundancy	6.3 (5.6)	11.0 (7.4)	10.9 (3.8)
Refinement			
Resolution (Å)	53.76‐1.47	53.68‐1.51	53.15‐1.35
No. reflections	377 315 (16 138)	61 149 (18 952)	793 975 (10 267)
*R*_work_/*R*_free_	0.198/0.228	0.196/0.226	0.181/0.193
No. atoms			
Protein	2887	2772	2797
Ligand/other	32/92	34/58	32/58
Water	150	117	150
*B*‐factors (Å^2^)			
All atoms	23	27	23
Ligand/inside pocket	24	39	30
Ligand/outside pocket	26	38	30
R.m.s. deviations			
Bond lengths (Å)	0.008	0.007	0.006
Bond angles (°)	0.9	0.9	0.9

a Values in parentheses are for highest resolution shell.

### 2.4 Thermal shift assay

For the thermal shift assay, 5 μg of Notum protein in 25 µL assay buffer (10 mmol/L Hepes, pH 7.4, 150 mmol/L NaCl and 6 × SYPRO Orange dye, Thermo Fisher Scientific) was mixed with 25 µL of compounds in buffer containing 2% DMSO. The samples were placed in a semi‐skirted 96‐well PCR plate (4‐Titude), sealed and heated in an Mx3005p qPCR machine (Stratagene, Agilent Technologies) from room temperature at a rate of 1°C/min for 74 cycles. Fluorescence changes were monitored with excitation and emission wavelengths at 492 and 610 nm, respectively. Reference wells, that is solutions without compounds but with the same amount of DMSO, were used to compare the melting temperature (Tm).

### 2.5 Notum inhibition assay

The fluorescent lipase substrate OPTS (8‐octanoyloxypyrene‐1,3,6‐trisulfonate) was dissolved in water at a concentration of 20 µmol/L. Chemical compounds were diluted into the 2 × enzyme assay buffer (20 mmol/L Hepes, pH7.4, 300 mmol/L NaCl) with purified active Notum at a concentration of 1 nmol/L. Each of the compound solutions was mixed with substrate at a 1:1 ratio to a final volume of 100 µL in a 96‐well flat‐bottom black polystyrol microplate, and fluorescence values were recorded in the Tecan Infinite F200 plate reader with an excitation wavelength of 435 nm (bandwidth 20 nm) and emission wavelength of 535 nm (bandwidth 25 nm). The inhibition curves are fitted with a one‐site model.

## 3 RESULTS

### 3.1 Crystallographic fragment screening indicates Notum‐melatonin binding

**Figure 1:**
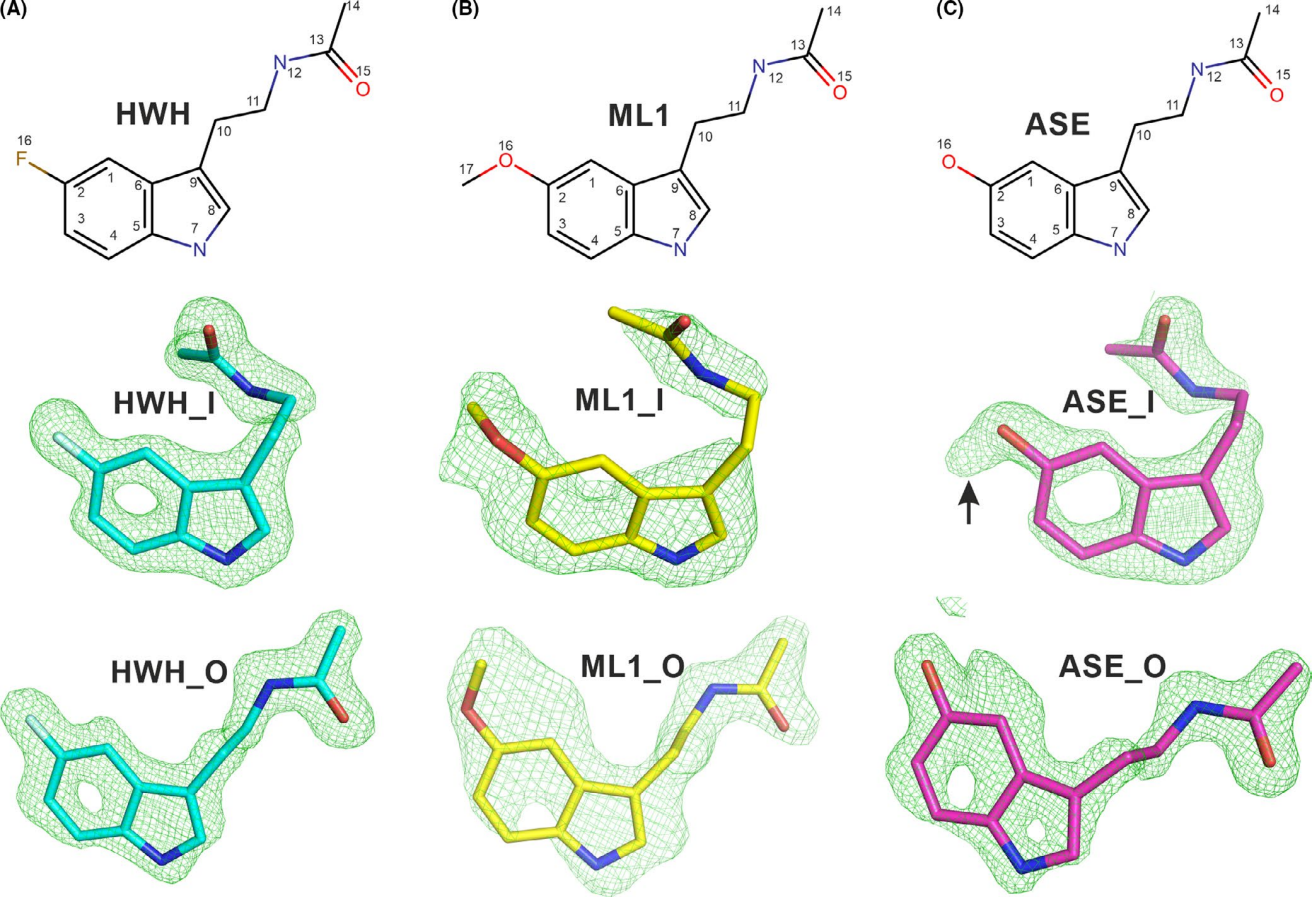
Chemical structures of Notum inhibitors and their electron density maps. The top row shows the inhibitor chemical structures; the middle row shows the ligands inside the Notum enzymatic pocket in sticks with |Fo–Fc| omit electron density maps contoured at 3 σ; the bottom row shows the ligands at the outside of the pocket. A, Fragment hit compound N‐[2‐(5‐fluoro‐1H‐indol‐3‐yl)ethyl]acetamide with PDB ligand name HWH (PDB code 6TR7). B, Melatonin with PDB ligand name ML1(PDB code 6TR5). C, N‐acetylserotonin with PDB ligand name ASE (PDB code 6TR6). Extra electron density associated with the C2 hydroxyl group of ASE_I, but not with ASE_O, is indicated by black arrow

**Figure 2:**
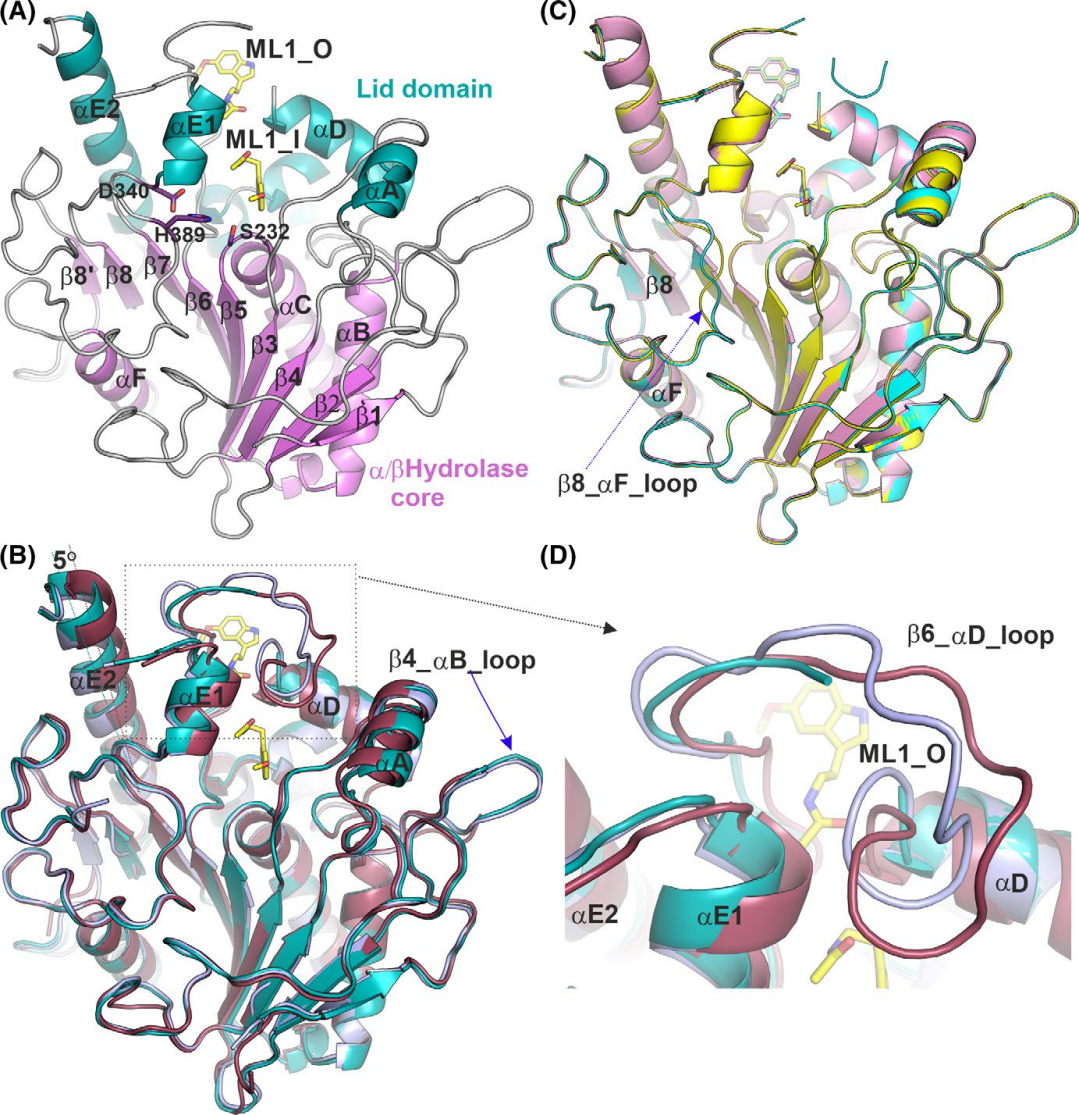
Cartoon representations of Notum structures. A, Notum‐melatonin complex structure. The conserved α/β‐hydrolase core structures are coloured in violet, the lid domain in teal and loops in grey. Melatonin is shown as yellow sticks. The enzyme catalytic triad side chains are shown as violet sticks. B, Alignment of the Notum‐melatonin complex (PDB code 6TR5), in teal colour) with two apo structures; one in the open conformation (PDB code 4UZ1, in light blue) and one in the closed conformation (dark red; PDB code 4UYU). C, Alignment of structures of Notum bound to melatonin (yellow; PDB code: 6TR5), N‐[2‐(5‐fluoro‐1H‐indol‐3‐yl)ethyl]acetamide (cyan; PDB code 6TR7) and N‐acetylserotonin (magenta; PDB code: 6TR7). D, Close up of the β6_αD loop region from B showing movement or disorder in apo and melatonin‐bound structures

**Figure 3:**
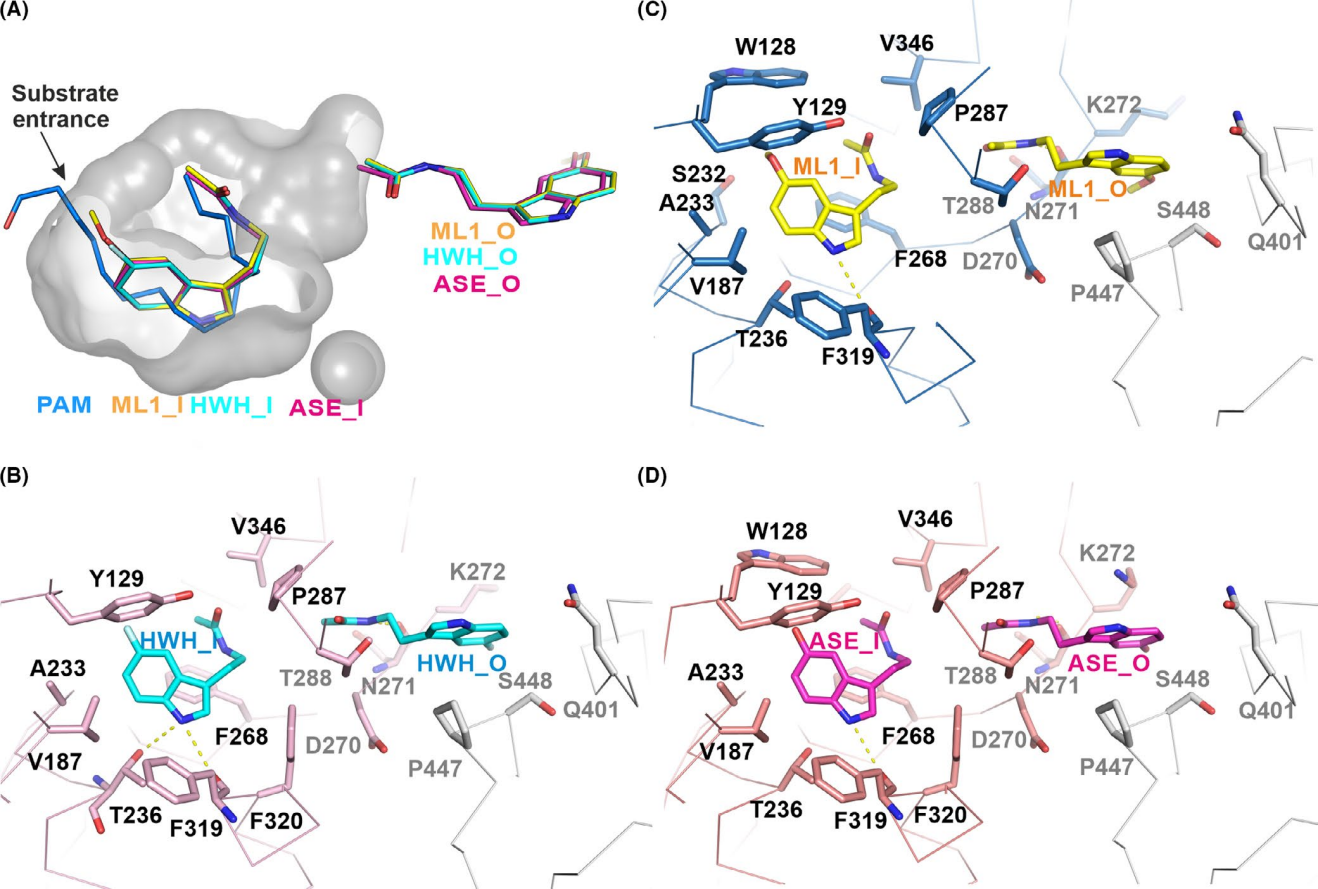
Notum enzymatic pocket and inhibitor binding details. A, The enzyme pocket is shown as a grey surface with 50% transparency. The bound ML1 (yellow; PDB code: 6TR5), HWH (cyan; PDB code: 6TR7) and ASE (magenta; PDB code: 6TR6) and palmitoleate (blue; PDB code 4UZQ) are shown as sticks. The outside pocket inhibitors are also shown. B‐D, The binding details of the Notum inhibitors. The Notum structure is shown as a ribbon with a symmetry‐related molecule in grey. The Notum residues interacting with enzymatic pocket inhibitors are labelled in black while the outside ones are in grey. Hydrogen bonds are shown as dashed yellow lines

In order to identify hit compounds for the development of novel Notum inhibitors, we used an X‐ray crystallography‐based fragment screen implemented at the XChem platform of Diamond Light Source (as described in Materials and Methods). The highly automated XChem platform, in combination with synchrotron beamline I04‐1, enabled us to efficiently screen 768 compounds from a fragment library for binding to Notum. Compounds were individually soaked into crystals of Notum and a high‐resolution (typically 1.8 Å) structures were determined for each of these putative Notum‐fragment complexes. The electron density maps provided evidence of compound binding associated with the active site of Notum for 61 of the crystal structures. Among the hits identified by this high throughput strategy, we were interested to note that one, N‐[2‐(5‐fluoro‐1H‐indol‐3‐yl)ethyl]acetamide (fragment 106, PDB ligand code: HWH, Figure 1A), is structurally very similar to melatonin. Melatonin only differs from HWH at the C2 position of the indole ring where a methoxy group replaces the fluoride atom of HWH (Figure 1A,B). This similarity led us to the notion that melatonin may also bind Notum and potentially inhibit its enzyme activity. To test this idea, we performed a Notum crystal soaking experiment using melatonin, collected high‐resolution X‐ray diffraction data and determined the crystal structure of the Notum‐melatonin complex at 1.51 Å resolution. The structure shows well‐defined electron density for two melatonin molecules bound to Notum (Figure 1B, melatonin PDB ligand code: ML1). The Notum‐HWH complex also contains two copies of HWH, and the positions and orientations of the two bound melatonin molecules are similar to those of the HWH. One melatonin molecule is located inside the enzymatic pocket (ML1_I, Figures 1B, 2A and 3A), while the other is positioned at the outer edge of the pocket away from the enzyme's catalytic triad (ML1_O, Figures 1B, 2A and 3A). We also used the crystal soaking approach to assay the Notum binding properties of N‐acetylserotonin, a precursor of melatonin, which has a hydroxyl group at the C2 position of the indole ring instead of the fluoride atom of HWH. As expected, the 1.35 Å resolution crystal structure showed that N‐acetylserotonin (PDB ligand code: ASE) also binds Notum. We observed well‐defined electron density for two ASE molecules (Figure 1C), which bind similarly to melatonin and HWH (Figure 2B, Figure 3). For all three complexes, the side chains of the compounds bound inside the enzymatic pocket are twisted at position C11 and have relatively less well‐defined electron density for the C10‐C11 bonds, especially in the melatonin (ML1_I) and N‐acetylserotonin (ASE_I) structures. The side chains of the inhibitors situated outside the pocket are extended (Figure 1, bottom row) and have somewhat better‐defined electron density.

### 3.2 The structural characteristics of compound‐bound Notum complexes

Notum adopts the “canonical” α/β‐hydrolase superfamily protein fold.^45^ It has a conserved core structure comprising an 8‐stranded β‐sheet protected by α‐helices and loops on both sides (Figure 2A, the core domain coloured violet, includes the β1‐β8 strands and the αB, αC and the αF helices). The Wnt deacylase Notum, like lipases, has a lid domain (Figure 2A, coloured in teal) comprising the αA, αD and αE helices and a β6‐αD loop. A moveable lid domain is a distinctive feature of lipases.^45^ To investigate if the Notum lid domain is also moveable, we aligned the melatonin‐bound Notum structure to the published Notum Apo and natural substrate‐bound structures. All the previously reported apo and substrate palmitoleate‐bound structures are in a closed conformation, with one exception (PDB code 4UZ1) which is in an open conformation, similar to the three compound‐bound structures reported here. As shown in Figure 2B, we aligned the melatonin‐bound Notum structure (cartoon in teal colour) with two apo structures, one in a typical closed conformation (PDB code 4UYU, in magenta) and the other is in an open conformation (PDB code 4UZ1, in pale blue). The melatonin‐bound structure superposes well with the open apo structure, with an rmsd of 0.52 Å for all 345 Cα atoms. When the melatonin‐bound structure is compared with the closed conformation, the core domain superposes well, with 202 out of 220 Cα atoms aligned with an rmsd of 0.4 Å. However, the lid domain helices (αA, αD, αE1 and αE2) in the melatonin‐bound structure are all shifted relative to the catalytic triad of the enzymatic pocket (Figure 2B). For example, the αE2 helix rotates 5° away from the catalytic centre around its C‐terminal end such that the N‐terminal end of the helix has a 3 Å shift in position (Figure 2B).

When all three compound‐bound structures reported here were aligned, all showed the same open conformation (Figure 2C). In lipases, the open conformation is active, with the active site fully available for substrate binding. As an open apo Notum structure has been reported (PDB code 4UZ1), it is not possible to distinguish if compound binding contributes to the formation or stabilization of the open conformation, or if the inhibitors only bind to the open conformation. In all three inhibitor‐bound structures, the β6‐αD loops are disordered (Figure 2C,D), while they are ordered in apo structures (although exhibiting plasticity) (Figure 2D). It is possible therefore that the disorder in these loops is caused by the compounds binding; however, we cannot exclude this being the result of the soaking procedure.

The Notum catalytic triad comprises three residues, S232, D340 and H389. S232 serves as a nucleophile, located in a sharp turn between β5 strand and αC helix (Figure 2A). This conserved structural element has been termed the “nucleophilic elbow” and comprises a consensus hydrolytic sequence G‐X‐Nu‐X‐G (X, any residue; Nu, nucleophile). The nucleophilic elbow region in Notum contains a characteristic Ramachandran plot unfavourable residue, G127. The G127‐W128 amide groups participate in the formation of the oxyanion hole in addition to the canonical S232‐A233 and G126 amides, thereby providing optimal stabilization during the transition state.

Next to the catalytic triad, there is a large hydrophobic Notum enzyme pocket where the enzyme‐substrate palmitoleic acid (PAM) has been shown to sit (Figure 3A). The nucleophile (S232) sits close to the acid group of the palmitoleate where it is attached to the Wnt serine residue. Alignment of the Notum‐melatonin structure with the palmitoleate‐bound structure (PDB code 4UZQ) indicates the methoxy group of ML1_I is bound close to the substrate entrance, near the palmitoleate acid group. The indole ring overlaps with most of the central part of the palmitoleate around the kinked *cis* double bond (C9‐C10), and the N‐acetyl group is close to the acyl tail (Figure 3A). The melatonin precursor N‐acetylserotonin (ASE) and the fragment hit (HWH) bind similarly as melatonin (Figure 3).

The melatonin‐Notum interactions are mainly hydrophobic (Figure 3C). ML1_I interacts with W128, Y129, V187, S232, A233, T236, F268, D270, P287, F319 and V346. Among these, S232 is the enzyme nucleophile residue, while W128, A233 form the oxyanion hole. The ML1_I indole ring mediates ring stacking interactions with F268 and Y129. The melatonin‐Notum interaction also involves hydrogen bond formation. The indole ring nitrogen atom (N7) of ML1_I forms a hydrogen bond with the main chain oxygen atom of F319 (Figure 3C), while the N‐12 atom of ML1_O forms a hydrogen bond with the main chain oxygen atom of N271. The ML1_O also interacts with D270, N271, T288 and K272 and is further stabilized by residues Q401, P447 and S448 from a symmetry‐related molecule in the crystal lattice (Figure 3C). Similar interactions with the fragment hit compound HWH and N‐acetylserotonin (ASE) are present for the majority of these Notum residues (Figure 3B,D). HWH_I does not form hydrophobic interactions with W128 and the nucleophile S232; however, it gains a hydrophobic interaction with F320 and a hydrogen bond between the T236 side chain and the HWH indole ring nitrogen (N7). ASE_I also does not interact with nucleophile S232 (Figure 3D), indicating the melatonin methyl group may be important for the interaction with S232. Like HWH_1, ASE_I also gains a hydrophobic interaction with F320.

### 3.3 Measurement of inhibitor potency

**Figure 4:**
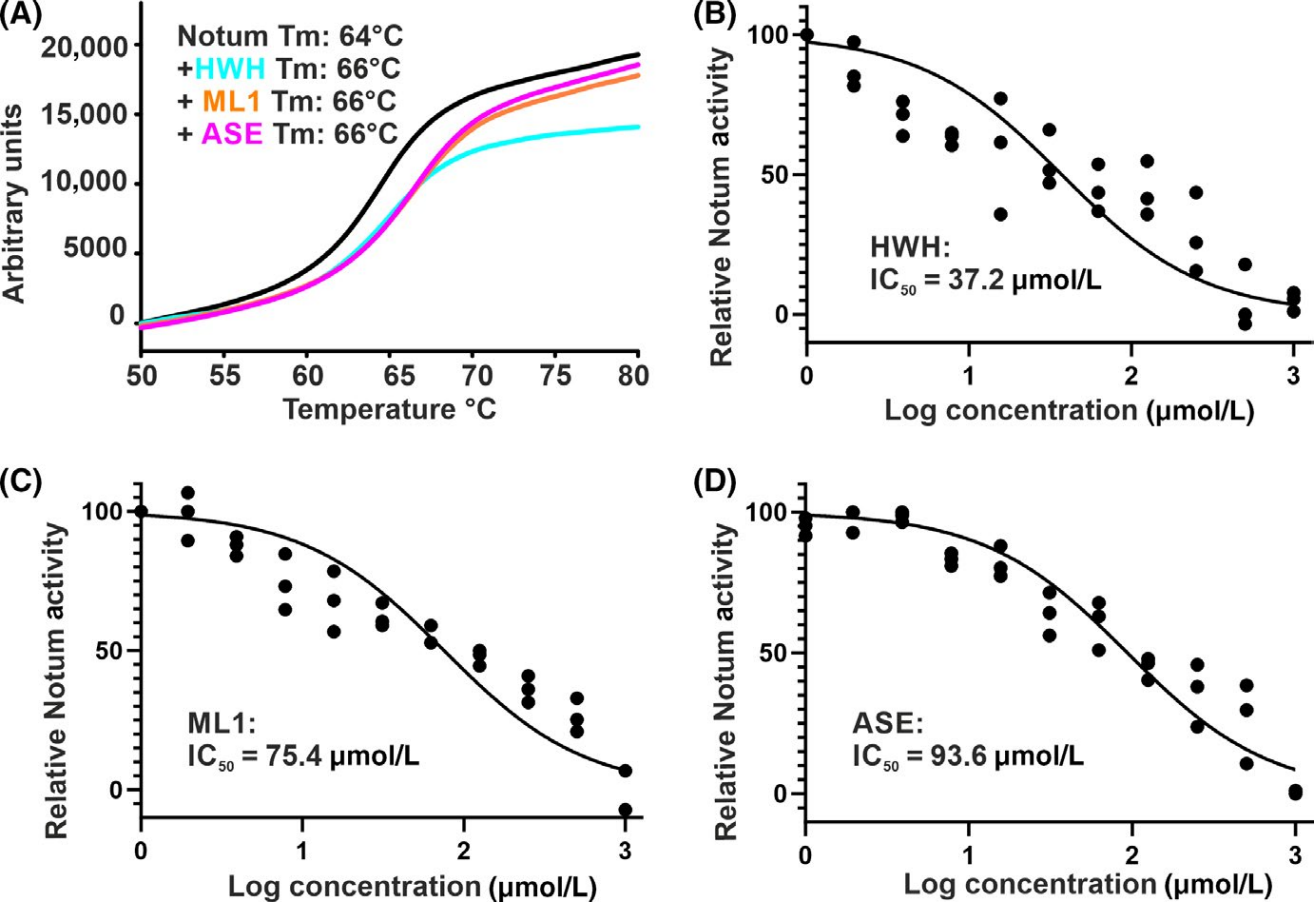
Thermal shift and enzymatic activity assays. A, Example of melting curves with compounds at 100 µmol/L concentration. The melting temperature (Tm) for Notum with each compound is indicated. B‐D, Notum enzymatic assay with OPTS as substrate. The curves were fitted with nonlinear regression (log inhibitor concentration verses normalized response)

To gain additional biochemical evidence of melatonin binding to Notum, we performed a thermal shift assay (also known as differential scanning fluorimetry). A Notum protein melting curve is shown in Figure 4A. Apo Notum protein (in the presence of 2% DMSO, the same amount used in the compound tests) shows a melting temperature (Tm) of 64°C. All the inhibitors stabilize Notum and increase the Tm to 66°C. It is widely accepted that ∆Tm 2°C or above is significant for indication of a small molecule binding to a protein.^46^ To further test the potency of the inhibitors, we performed in vitro enzyme inhibitory assays. The Notum enzyme can act on a common lipase fluorescent substrate OPTS (8‐octanoyloxypyrene‐1,3,6‐trisulfonate). OPTS is not a natural Notum‐specific substrate; however, with purified enzyme, it can be used to measure enzyme activity quantitatively^32^ and is similar to the octanoyl lipid‐linked p‐nitrophenyl (pNP8) substrate previously used for the measurement of the Km value of 12.6 µmol/L to Notum.^29^ In each of our crystal structures, two inhibitor molecules were observed: one within the enzyme pocket with interacting residues all within one protein molecule and the other stabilized by a neighbouring protein molecule through crystal packing. Thus, the inhibitor molecule bound on the enzyme surface is less likely to contribute to the IC_50_ measured in solution. We therefore fitted the inhibition curve with a one‐site model. As shown in Figure 4, melatonin (ML1) can inhibit Notum with an IC_50_ of 75 µmol/L, while the fragment hit compound HWH shows higher potency (IC_50_ of 37 µmol/L). N‐acetylserotonin is less potent (IC_50_ of 94 µmol/L). Overall, the observed potencies of these inhibitors are in the middle range of all the fragment hits identified, which are relatively weak. Structure‐guided design could dramatically improve the potency of these hits, as exemplified by the work in which IC_50_ of an unrelated fragment from our screen has been improved from 33 to 0.032 µmol/L.^33^

## 4 DISCUSSION

**Figure 5:**
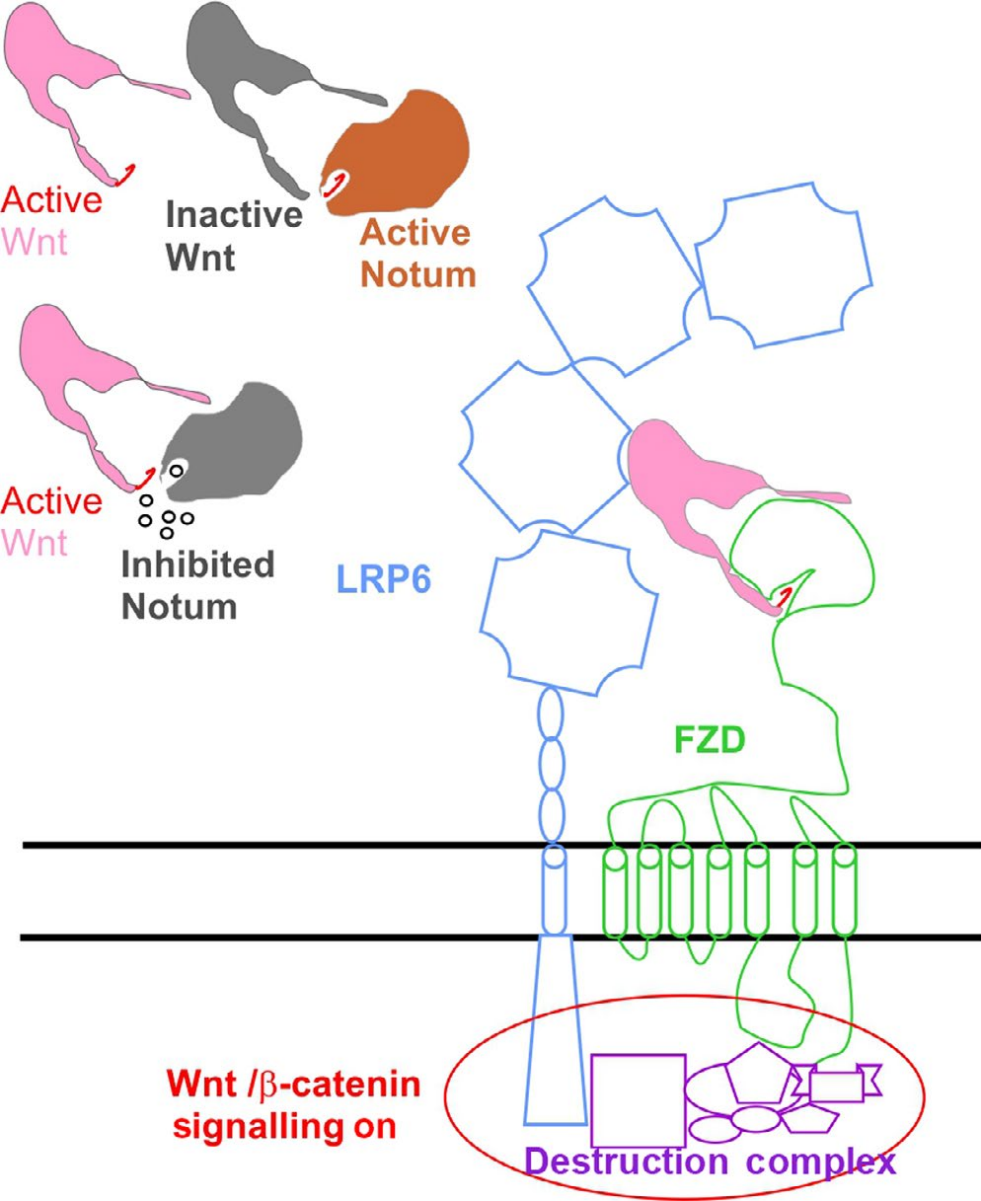
Cartoon diagram showing model for Wnt signalling modulation by Notum small molecule inhibitors. The small molecule inhibitors are represented as small circles. The active Wnts are shown in pink colour with palmitoleate lipid in red and inactive Wnt in grey. The active Notum is in orange and inactive Notum in grey

The Wnt/β‐catenin signalling pathway is composed of core components including Wnt ligands, Frizzled receptors and low‐density lipoprotein receptor‐related protein (LRP5/6) co‐receptors, as outlined in Figure 5. Notum is a Wnt ligand deacylase, which removes the functionally essential palmitoleate moiety.^29^ Notum inhibitors, such as those described here, can occupy the enzyme pocket and inhibit enzyme activity, thus leaving more active Wnt ligands available for receptor engagement (Figure 5). Once the Wnt receptor and co‐receptor are engaged, the complex recruits and suppresses the function of the cytoplasmic β‐catenin “destruction complex” consisting of glycogen synthase kinase 3β (GSK3β), casein kinase 1α (CK1α), the scaffold protein AXIN and the tumour suppressor adenomatous polyposis coli (APC). When the “destruction complex” is disabled, β‐catenin is no‐longer degraded and accumulated nuclear β‐catenin modulates transcription of target genes. Modulation of Wnt signalling by inhibiting Notum offers an opportunity to target situations when Wnt signalling upregulation may be beneficial, such as Alzheimer's disease and osteoporosis. Our observation that melatonin directly binds to Notum and inhibits its activity suggests a molecular mechanism for previous reports that melatonin can activate Wnt/β‐catenin signalling in neuron cells and osteoblasts.^23^, ^24^, ^25^, ^47^

We report here a structural similarity between melatonin and the Notum inhibitor HWH identified from an X‐ray crystallographic‐based fragment screen. This observation suggested that melatonin could target Notum. We then showed that melatonin and its precursor N‐acetylserotonin indeed can bind Notum and inhibit its enzyme activity. As melatonin is naturally produced in the brain and can access all tissues, the crystal structure of the Notum‐melatonin complex may provide valuable information for the design of more potent brain accessible drugs which may be useful for neurodegenerative disease. Interestingly, melatonin itself has already been demonstrated to be neuroprotective in ageing and AD animal models, as its administration decreases the accumulation of Aβ and hyperphosphorylated tau.^48^, ^49^, ^50^ Activation of Wnt signalling can directly inhibit the “destruction complex” component GSK3β and thus decrease tau hyperphosphorylation.^51^ The observation that melatonin can target Notum may provide an additional mechanism for melatonin's neuroprotective role in Alzheimer's disease. However, the IC_50_ value (75 µmol/L) from our in vitro enzyme assay is considerably higher than a previously reported concentration of 2 µmol/L that was sufficient for neuron cell β‐catenin activation,^23^ suggesting the role of melatonin in Wnt signalling may be more complex. It is well established that melatonin mainly acts on its high‐affinity G protein‐coupled receptors, MT1 (MTNR1A) and MT2 (MTNR1B), while a third receptor MT3 (MTNR1C) may be quinone reductase 2.^52^, ^53^, ^54^ It is not known if activation of these receptors could contribute to Wnt signalling upregulation.

**Figure 6:**
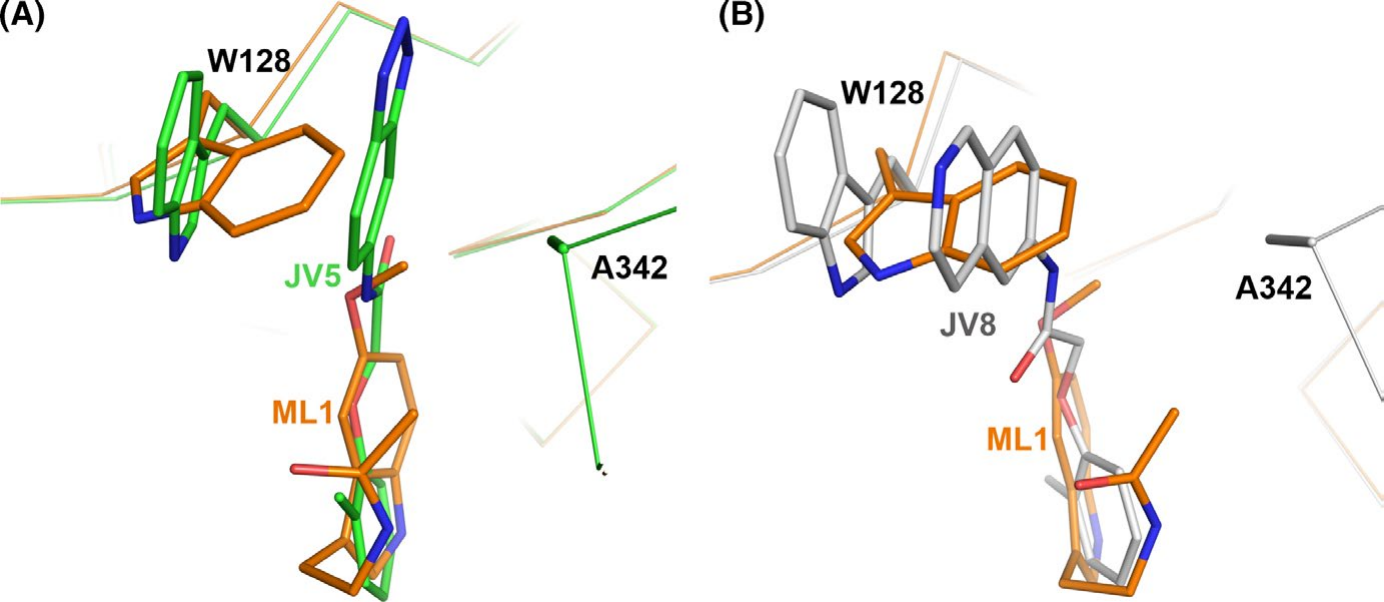
Comparison of the binding mode of melatonin with JV5 and JV8. A, The binding mode of melatonin (brown; PDB code 6TR5) is overlaid with indazole 38, JV5 (green sticks, PDB code 6R8Q) and B, with isoquinoline 45, JV8 (grey sticks, PDB code 6R8R). The main chain of Notum is shown as Cα trace and side chains of residues W128 and A342 as sticks

Both melatonin and N‐acetylserotonin have protective roles in brain injury, liver damage and bone health.^15^, ^17^ The Notum protein is expressed in bone,^30^ liver^55^ and brain.^56^ Despite both melatonin and N‐acetylserotonin being very weak Notum inhibitors, the structural information provided here forms a solid basis for the development of potent Notum inhibitors. We recently reported such a development in which two inhibitors, indazole 38 and isoquinoline 45, modified from initial hits had their potency increased up to 100‐fold.^33^ In order to investigate how melatonin could be modified to increase its potency, we superposed our melatonin‐bound structure with the bound indazole 38 (JV5, PDB code 6R8Q) and isoquinoline 45 (JV8, PDB code 6R8R), as shown in Figure 6. The potency of melatonin may be enhanced by adding an indazole or isoquinoline ring to the methoxy group to create ring stack interactions with the side chain of residue W128. Ultimately, rational drug design based on this structural information may lead to the development of clinically useful drugs for the treatment of neurodegenerative diseases and osteoporosis.

### 4.1 Accession numbers

The structure factors and coordinates of the complexes of Notum‐melatonin, N‐acetylserotonin and N‐[2‐(5‐fluoro‐1H‐indol‐3‐yl)ethyl]acetamide have been deposited in the Protein Data Bank under identification codes 6TR5, 6TR6 and 6TR7, respectively.

## CONFLICT OF INTEREST

The authors have no competing financial interests to declare.

## AUTHOR CONTRIBUTIONS

YZ and EYJ designed the project and wrote the manuscript together with JH and JR, MJ and WL. YZ performed experiments and analysed data with JR. WL helped with tissue culture.
